# Diversity of actin architecture in human osteoclasts: network of curved and branched actin supporting cell shape and intercellular micrometer-level tubes

**DOI:** 10.1007/s11010-017-3004-2

**Published:** 2017-03-14

**Authors:** Paula Pennanen, Maria Helena Alanne, Elnaz Fazeli, Takahiro Deguchi, Tuomas Näreoja, Sirkku Peltonen, Juha Peltonen

**Affiliations:** 1 Department of Cell Biology and Anatomy, Institute of Biomedicine, University of Turku, Kiinamyllynkatu 10, 20520 Turku, Finland; 2 Laboratory of Biophysics, Department of Cell Biology and Anatomy and Medicity Research Laboratories, University of Turku, P.O. Box 123, 20521 Turku, Finland; 3 Division of Pathology, Department of Laboratory Medicine, Karolinska Institutet, Stockholm, Sweden; 4 Department of Dermatology, University of Turku and Turku University Hospital, PO BOX 52, 20521 Turku, Finland

**Keywords:** Osteoclasts, Branched actin, Curved actin, Micrometer-level tubes, MLT, Tunneling nanotubes, TNT, STED microscopy, Nuclear transport

## Abstract

Osteoclasts are multinucleated bone-resorbing cells with a dynamic actin cytoskeleton. Osteoclasts are derived from circulating mononuclear precursors. Confocal and stimulated emission depletion (STED) super-resolution microscopy was used to investigate peripheral blood-derived human osteoclasts cultured on glass surfaces. STED and confocal microscopy demonstrated that the actin was curved and branched, for instance, in the vicinity of membrane ruffles. The overall architecture of the curved actin network extended from the podosomes to the top of the cell. The other novel finding was that a micrometer-level tube containing actin bridged the osteoclasts well above the level of the culture glass. The actin filaments of the tubes originated from the network of curved actin often surrounding a group of nuclei. Furthermore, nuclei were occasionally located inside the tubes. Our findings demonstrated the accumulation of c-Src, cortactin, cofilin, and actin around nuclei suggesting their role in nuclear processes such as the locomotion of nuclei. ARP2/3 labeling was abundant at the substratum level of osteoclasts and in the branched actin network, where it localized to the branching points. We speculate that the actin-containing tubes of osteoclasts may provide a means of transportation of nuclei, e.g., during the fusion of osteoclasts. These novel findings can pave the way for future studies aiming at the elucidation of the differentiation of multinucleated osteoclasts.

**Electronic supplementary material:**

The online version of this article (doi:10.1007/s11010-017-3004-2) contains supplementary material, which is available to authorized users.

## Introduction

Bone dynamics refers to the continuous process of replacing old bone with new. Osteoclasts function as bone-resorbing cells thus contributing to the catabolic component of bone dynamics. Multinuclear osteoclasts are derived from circulating mononuclear precursor cells, which fuse to form osteoclasts [1–3]. The differentiation process of osteoclasts is facilitated by receptor activator of nuclear factor kappa-B ligand (RANKL) and macrophage colony-stimulating factor (M-CSF) [4, 5].

The classic hallmarks of osteoclasts are related to resorption and the subcellular distribution of actin. Cultured osteoclasts develop specialized adhesion structures called podosomes which have F-actin cores. During differentiation, podosomes are organized into podosome belts surrounded with a loose F-actin cloud at the cell periphery [6]. On bone, osteoclasts form one of the hallmarks, the sealing zone that brings cell membrane in close contact with the bone surface delineating the resorption area between osteoclast and bone [7, 8]. The dense actin ring structure of the sealing zone has been used as a marker for active, resorbing osteoclasts [9, 10].

Actin cytoskeleton of osteoclasts is a dynamic structure which undergoes rapid changes during cell migration, fusion, and resorption [7, 11]. Actin contributes to the mechanical properties of the cytoskeleton by forming linear stress fibers [12]. Actin has a role in several cellular processes such as vesicular trafficking and formation of cellular extensions. Actin is involved in variable cellular structures such as lamellipodia, filopodia, tunneling nanotubes (TNTs), podosomes, and actin patches [13–15]. Tunneling nanotubes are F-actin-rich structures, which bridge two different cells above the substratum and are enclosed with membrane that continues with the membranes of connected cells [14]. These intercellular structures are essential for the fusion of mononuclear osteoclast precursors and the regulation of osteoclastogenesis. Various cellular components, such as small molecules, calcium ions, different proteins, mitochondria, and membrane vesicles, are transported through these intercellular bridges [16, 17].

A new aspect of actin research has revealed that actin has a crucial role in many nuclear processes such as RNA transcription and processing, nuclear transport, and chromatin remodeling. Most of the nuclear actin is in a monomeric form, but low amounts of F-actin exist. Nuclear actin contains more than 30 actin-binding proteins like cofilin, CapG (macrophage-capping protein), and MAL (T-Cell differentiation protein). Actin may also interact with nuclear membrane proteins such as enaptin and protein 4.1 [18, 19]. The connection between cytoplasmic and nuclear actin is still unknown.

As demonstrated by ex vivo studies, curved actin and its semi-flexible properties have been the recent focuses of actin research. Factors such as actin-binding proteins, compression, and temperature can modulate actin filaments. Compression leads to energy storage in a curved actin network [20–22]. Tyrosine kinase c-Src regulates rearrangement and remodeling of actin filaments via interactions, such as cortactin. C-Src also plays an important role with the amount of actin and reorganization of podosomes [23, 24]. The detailed mechanism of how c-Src acts on actin is still unclear. However, c-Src is known to be involved in the formation of actin-driven protrusions like lamellipodia, filopodia, and membrane ruffles where cortactin is also enriched [25]. Cortactin, a c-Src substrate, regulates the actin-binding protein ARP2/3 complex by stabilizing actin branches after they are formed and strengthens the direct interaction between ARP2/3 and F-actin. Actin is needed for cell motility, invasion, cellular protrusion formation, and membrane trafficking [26–28].

Polymerization and depolymerization of actin are needed for rapid remodeling of actin network. Cofilin severs actin filaments and increases the number of free ends where submits can be added. Cofilin contributes to the recycling of actin subunits and supports new filament growth. Cofilin-saturated filaments are proven to be even 20-fold more receptive in bending than bare filaments [29, 30].

A number of super-resolution imaging techniques have been developed during the last 10 years. The resolution of ordinary optical imaging/light microscopy is restricted to half of the wavelength of the light by diffraction. Super-resolution imaging enables analysis of the features of living organisms in unprecedented detail and to combine this structural information with its functional properties [31]. In the present study, confocal and STED microscopies were used to investigate peripheral blood-derived human osteoclasts cultured on a glass surface, which revealed the organization of actin and actin-containing intercellular tubes, which we referred to as micrometer-level tubes (MLTs).

## Materials and methods

### Human osteoclast, macrophage, and keratinocyte cultures

Human osteoclast cultures were established as described by Heervä et al. [32]. Briefly, mononuclear monocytes including osteoclast precursors were collected with Ficoll-Paque PLUS (GE Healthcare Bio-Sciences, Uppsala, Sweden) centrifugation from fresh blood samples from healthy volunteers. This study has been performed in accordance with the Declaration of Helsinki and approved by the Ethics Committee of Southwest Finland Hospital District. Participants gave their informed written consents to osteoclast cultures. The study was carried out at Turku University Hospital and the University of Turku. Cells were seeded on glass coverslips, half a million cells per glass (12 mm round coverslips with thickness of 0.17 mm *H* = 1.5, Marienfeld GmbH & Co.KG, Germany), and differentiated into multinuclear osteoclasts in a medium containing alpha-MEM (Gibco, Grand Island, NY), 10% heat-inactivated fetal bovine serum (Gibco), penicillin–streptomycin, 10 mM HEPES (Sigma-Aldrich), RANKL (20 ng/ml, Peprotech, Rocky Hill, NJ), and M-CSF (10 ng/ml, R&D systems, Minneapolis, MN) for 8–10 days.

To prepare the macrophage culture, monocytes were isolated from human peripheral blood samples by gradient centrifugation as above. Half a million cells per glass coverslip were seeded in a serum-free medium and allowed to settle for 2 h [33]. Non-adherent cells were washed away and the adherent cells were cultured for 7 days, and differentiated into macrophages in a medium containing alpha-MEM, 10% heat-inactivated fetal bovine serum, penicillin–streptomycin, and M-CSF.

Normal keratinocytes from healthy donors were cultured as described by Siljamäki et al. [34].

### Immunofluorescence

Osteoclast cultures were fixed with 4% paraformaldehyde in PBS and incubated for 15 min at room temperature. The cells were subsequently permeabilized with 0.1% Triton X-100 or 0.01% Tween-20 in PBS on ice for 5 min. To prevent non-specific binding, samples were preincubated in 1% BSA–PBS for 30 min. The following fluorochrome conjugates were used with 1:100 dilution in 1% BSA in PBS: ATTO 647-Phalloidin (Sigma-Aldrich, 65906), or Alexa Fluor^®^ 488 Phalloidin (Molecular Probes, Eugene, OR, A12379). Nuclei were visualized with a Hoechst 33342 (Molecular Probes, H3570) with a 1:10 000 dilution. To study the potential binding partners of actin, the cells were double labeled for actin and c-Src (Cell Signaling, 2109), cortactin (Santa Cruz, 55579), anti-arp2 antibody (Abcam, ab47657), or cofilin (Santa Cruz, 53934). For secondary antibodies, Alexa Fluor 488 goat anti-mouse IgG (Abcam, Cambridge, UK) and Alexa Fluor 488 goat anti-rabbit IgG (Molecular Probes, OR, USA) antibodies were used. After immunolabeling, the coverslips were mounted with Mowiol (Sigma-Aldrich).

### Localization of actin and membrane ruffles

Osteoclast and macrophage cultures were permeabilized with a 0.01% Tween-20 in PBS on ice for 5 min after fixation with 4% PFA and blocked with 1% bovine serum albumin in PBS for 30 min. To visualize the membrane structure of the osteoclasts, the cells were stained with 5 µM DiI (DiI Stain: 1,1′-dioctadecyl-3, 3,3′,3′-tetramethylindocarbocyanine perchlorate, Life Technologies) in 1% BSA in PBS. Nuclei of osteoclasts and macrophages were stained with Pico Green (Life Technologies) in a 1:10,000 dilution in 1% BSA in PBS, and for actin staining phalloidin conjugated with STAR635 (Abberior GmbH, Gottingen, Germany) was used with a 1:100 dilution in 1% BSA in PBS for 1 h. Subsequently, cells were washed with PBS three times, each time for 5 min and placed in PBS. Images were taken with a confocal Leica TCS SP5 STED microscope (Leica Microsystems GmbH, Mannheim, Germany) and analyzed with ImageJ software (version of 1.49p).

### Fluorescence imaging

Images were acquired using a Leica TCS SP5 STED microscope, equipped with MaiTai HP (Leica Microsystems GmbH, Mannheim, Germany and Spectra-Physics, US) and operated with LAS AF software (Leica Microsystems). Phalloidin Atto647N or Star635 was excited by a 635 nm pulsed laser (LDH-P-C-640B, PicoQuant, Berlin, Germany), and fluorescence was collected with an avalanche photo diode (APD) detector at a 665–705 nm range. PicoGreen or Alexa Fluor^®^ 488 Phalloidin was excited at 488 nm and detected in 500–560 nm by photomultiplier tubes. DiI was excited with 561 nm and detected in 570–610 nm by photomultiplier tubes. For super-resolution STED imaging, Atto647N or Star635 was depleted at 760 nm. Images of cells stained with DiI were acquired with a water immersion objective lens (N.A.1.2 63x water, Leica) and the remainder was acquired with an oil immersion objective lens (N.A.1.4 100x Oil, Leica). The confocal pinhole was set to one airy unit, with a line-scan speed of 600 Hz, a line averaging of either 8 or 16. The pixel size (sampling) was set to 20 nm for STED imaging, thus satisfying the Nyquist sampling requirement [35]. STED and confocal images were deconvolved with ImageJ 1.49 software (National Institutes of Health) using 5 iterations of the Tikhonov–Miller deconvolution algorithm (Biomedical Image Group, EPFL, Switzerland) [36]. The snapshot images of 3D reconstructed volumes were taken with ImageJ 1.49 software. Additionally, images were further processed into collages with CorelDraw X7.

## Results

**Fig. 1:**
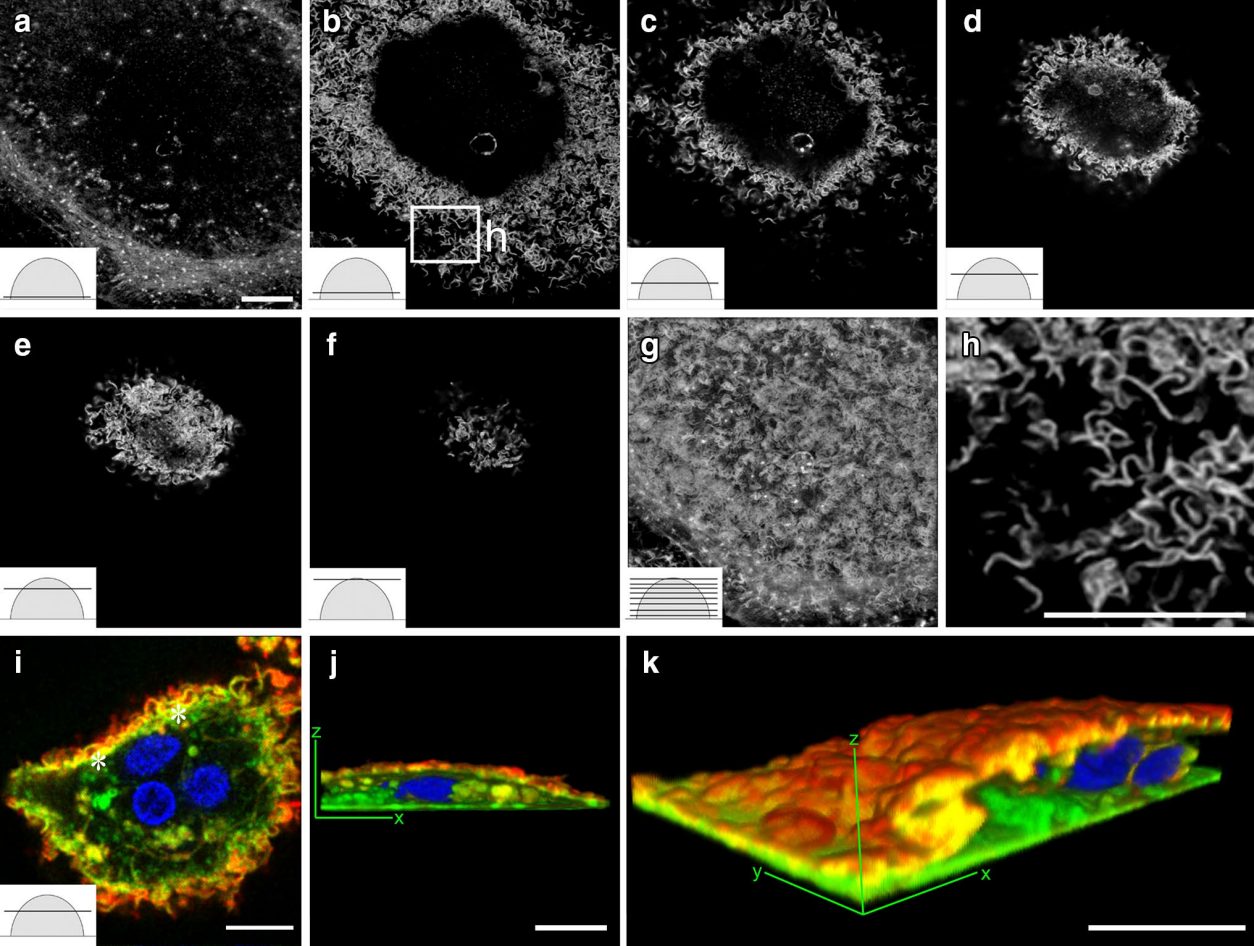
Actin displayed curved and branched architecture in cultured osteoclasts. Mature osteoclasts cultured on glass coverslips were labeled with phalloidin to visualize the actin cytoskeleton. The cells were imaged with STED microscopy from *bottom* to *top* (*insets*) (**a**–**h**). The phalloidin labeling for actin seen as *white spots* demonstrated scattered podosomes in the lowest optical sections (**a**). Super-resolution demonstrates the curved and branched actin (**b**–**f**). *Panels* **a**–**f** are shown as a merged image (**g**). The area in the *panel b box* is a demonstration of high-power magnification (**h**). A conventional confocal microscopy of an osteoclast labeled with actin (*green*), membrane lipids (*red*), and nuclei (*blue*) demonstrates the intimate association of actin and membrane ruffles (*yellow*) in the cell periphery (**i**–**k**). The two cross-sectional views demonstrate the 3-dimensional structure of an osteoclast (**j**–**k**). The schematic illustrations of an osteoclast represent a *semicircular shape* and the *line* indicates a cross-sectional level (Z-stack). *Scale bars* 10 µm. (Color figure online)

As demonstrated in Fig. 1, actin displayed curved and branched architecture in osteoclasts cultured on glass coverslips. Cells were optically sectioned with a STED microscope from bottom to top. Phalloidin labeling addressed scattered podosomes in the lowest optical sections (Fig. 1a). Actin morphology changed immediately above the lowest optical sections, and it started showing curved and branched actin structures. Curved and branched actin was found in roughly 60–80% of the osteoclasts, and visualized in each cross-sectional level from the bottom level to the apical sections. STED microscopy revealed improved resolution images of the curved and branched actin structure of osteoclasts compared to confocal microscopy (Fig. 1c, i). The overall architecture of the curved actin network extended from podosomes to the top of the cell. Conventional confocal microscopy of an osteoclast labeled for actin, membrane lipids, and nuclei demonstrated an intimate association of actin and membrane ruffles in the cell periphery (Fig. 1i–k). Some of the curved actin structures were strongly colocalized with membrane lipids (asterisks in Fig. 1i). This suggests that the curved actin structures are not only within the cell body but also extend into membrane protrusions on the cell surface. The snapshot images of 3D reconstructed volumes showed that membrane signals are much stronger at the cell periphery than the cell bottom (Fig. 1j–k).This allows us to speculate that protruding curved actin leads to an increase in the area of the surface membrane.

**Fig. 2:**
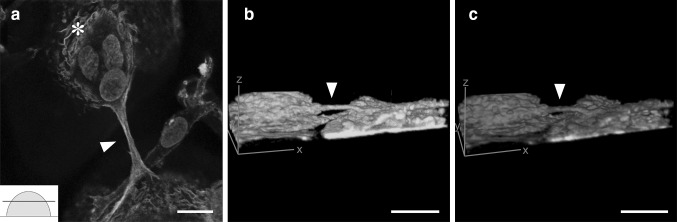
Thick, micrometer-level tubes bridge adjacent osteoclasts above the level of the culture substratum. Multinuclear osteoclasts were labeled for actin (*green*), membrane lipids (*red*), and nuclei (*blue*), and visualized with confocal microscopy (**a**–**c**). Phalloidin labeling visualized a thick protrusion (*arrowhead*) bridging adjacent osteoclasts above the level of cell culture substratum (*inset* in **a**). Clusters of nuclei were often surrounded by curved actin filaments (*asterisk* in **a**) which in turn were associated with the actin of the bridging tubes. **b** and **c** are 3-dimensional images which can be rotated in the supplement file to demonstrate the location of the bridging tube. *Scale bars* 10 µm

**Fig. 3:**
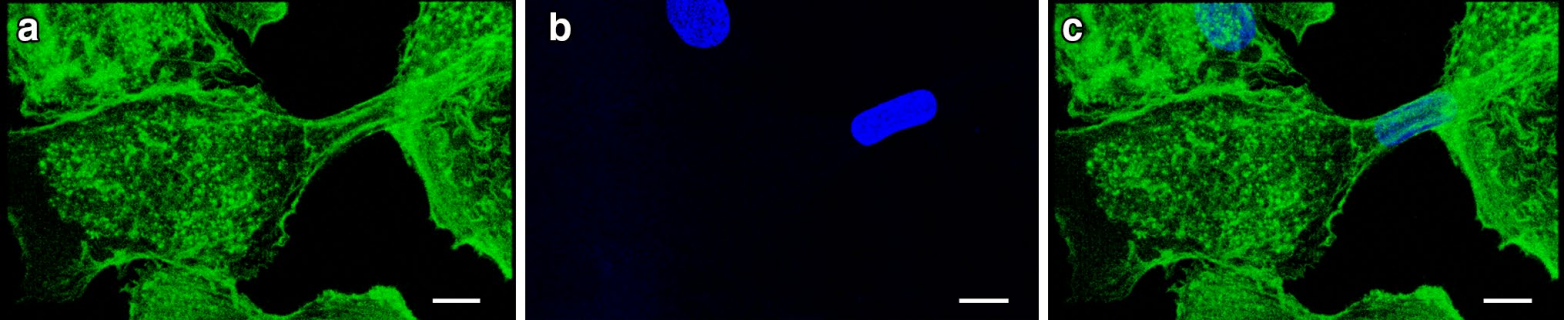
A nucleus is observed in a micrometer-level tube. Osteoclasts were labeled with actin (*green*) (**a, c**) and nuclei (*blue*) (**b**–**c**). *Scale bars* 10 µm

**Fig. 4:**
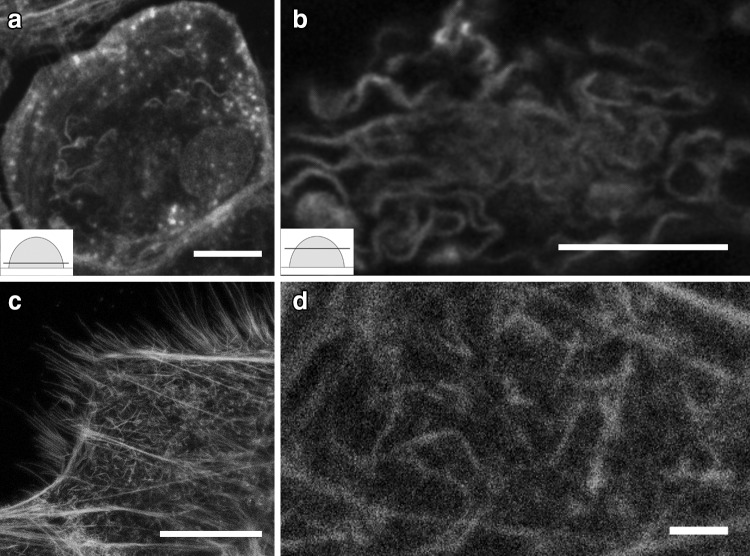
Curved and branched actin is observed in macrophages and keratinocytes. Human blood-derived macrophages on glass coverslips were labeled for actin (*green*) and nuclei (*blue*) and visualized with confocal microscopy. Podosomes were seen at the substratum level of the macrophages (**a**). The actin was curved and branched in the macrophages (**b**). In addition, human keratinocytes contained curved and branched actin (*green*) (**c**–**d**). *Scale bars* 10 µm (**a**–**c**) and 1 µm (**d**)

Another crucial detail is elucidated by the actin-rich intercellular structure in Fig. 2. Confocal microscopy visualized micrometer-level tubes, or MLTs, containing actin between two osteoclasts well above the level of the cell culture substratum (arrowhead in Fig. 2a, Supplementary video 1). These bridging protrusions had a diameter of ~1–5 µm and a length up to 40 µm. The actin filaments of the tubes were originated from the network of curved actin (Fig. 2a). The snapshot images of the reconstructed 3D volume clearly showed that the tube is above the substrate level (Fig. 2b). The membrane staining clearly showed that the MLT is enclosed within the membrane and that the membrane continued on to the membranes of the cells being bridged (Fig. 2c). In addition, the nuclei were often closely surrounded by the curved actin (asterisk in Fig. 2a), which in turn was associated with the actin of the bridging tubes. Furthermore, nuclei were occasionally seen inside the MLTs (Fig. 3). These nuclei showed elongated morphology while the intracellular nuclei were rounded (Fig. 3b). In order to elucidate whether curved and branched actin is specific to osteoclasts, we analyzed macrophages differentiated from monocytes without RANKL, and human keratinocytes from skin samples. The results showed that curved and branched actin was also present in macrophages and keratinocytes (Fig. 4).

**Fig. 5:**
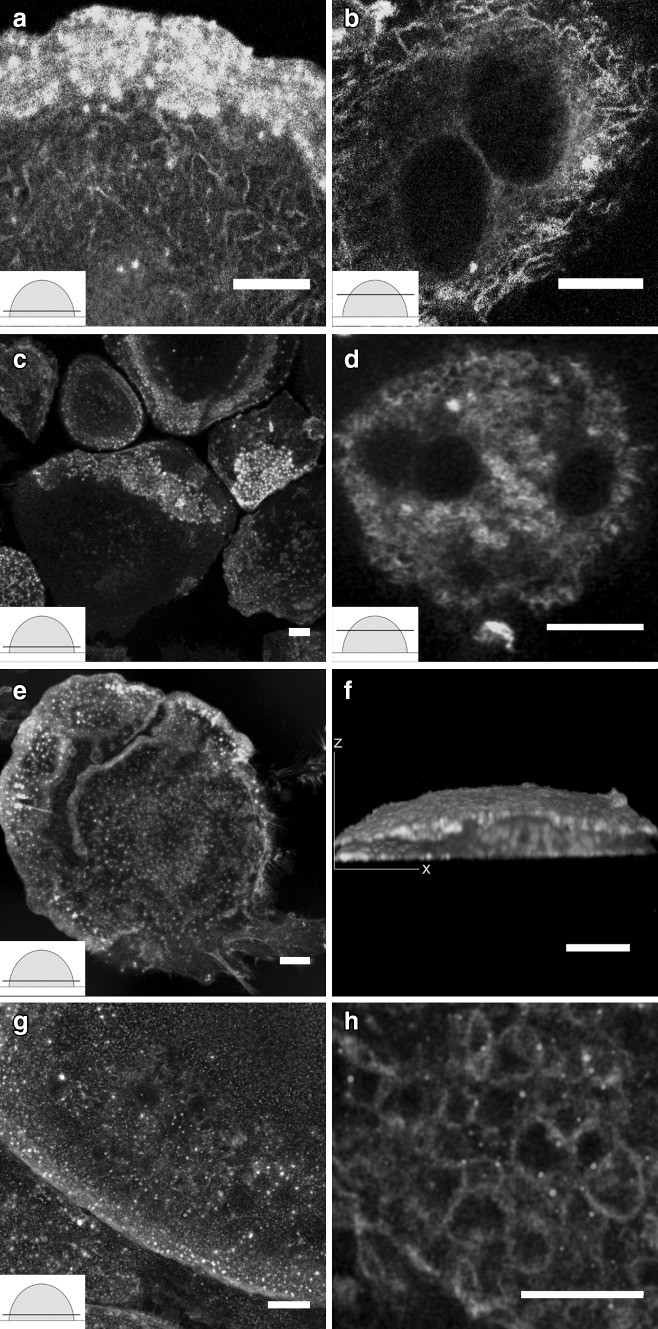
Actin co-labeled with c-Src (**a**–**b**), cortactin (**c**–**d**), cofilin (**e**–**f**), and ARP2/3 (**g**–**h**). Double labeling of osteoclasts with antibodies to actin (*green*) and c-Src (*red*) displayed partial colocalization in the bending actin at the periphery of the cell (**a**) and around the nuclei (**b**). Double labeling for actin (*green*) and cortactin (*red*) showed some colocalization at the cell periphery (**c**). Cortactin accumulated around nuclei (**d**). Cofilin (*red*) and actin (*green*) did not show colocalization; however, cofilin was found in the center of the cell near the nuclei (**e**–**f**). ARP2/3 (*red*) was abundant at the substratum level of osteoclasts (**g**) and was localized at the branching points of the actin network (*green*) (**h**). *Scale bars* 10 µm

Double labeling for c-Src and actin showed that c-Src was partially colocalized with the bending actin in the periphery of the cell and around the nuclei. Double labeling for cortactin and actin showed some colocalization in the cell periphery and cortactin accumulation around the nuclei. Cofilin assembled in the center of the cell nearby nuclei but did not colocalize with actin. ARP2/3 labeling was abundant at the substratum level of osteoclasts and in the branched actin network, where it localized to the branching points (Fig. 5).

## Discussion

Traditionally recognized actin-containing structures in osteoclasts cultured on glass include, e.g., the actin ring and podosome belt with an actin cloud. The actin cloud is composed of actin cables with adhesion molecules such as αvβ3 integrins and vinculin around the podosomes [6]. STED microscopy enabled us to discover novel characteristics of the diversity of actin architecture and its dynamic nature in osteoclasts. We have shown that curved and branched actin is situated under the membrane of the osteoclast extending from the podosomes at the substratum level to the top of the cell. The present study is the first to demonstrate curved actin filaments colocalized with membrane lipids in osteoclasts. Demonstration of curved actin in cultured macrophages and keratinocytes shows that this type of actin organization is not specific to osteoclasts. To date, curved actin has been studied under ex vivo conditions where mechanical force or actin-binding proteins cofilin and gelsolin have been shown to modulate the conformation of actin [22, 37–41]. Previous ex vivo studies have also shown that networks of actin can restore energy. These results also suggested that these phenomena might influence, e.g., cell shape and motility [42–44].

The present study demonstrates for the first time that MLTs bridge adjacent osteoclasts above the level of the culture substratum. Interestingly, a nucleus was occasionally visualized inside an MLT. The MLTs thus provide a potential passage way for the translocation of nuclei from one osteoclast to another. MLTs and TNTs showed two differences: the MLTs were much wider than the TNTs, and only one MLT was found per cell while the TNTs were more numerous. As actin filaments extend from MLTs to surround a group of nuclei, it is feasible to speculate that actin may influence the nuclear positioning within the cell and that these actin networks may help osteoclasts to adapt to rapid morphological changes, e.g., during cell fusion.

To further understand the relationship between actin and kinase c-Src, cortactin, cofilin, and ARP2/3, we localized these proteins during osteoclast differentiation on glass. We found that c-Src and cortactin were partially colocalized with the actin in the cell periphery suggesting that they may play a role in the rearrangement and stabilization of the curved and branched F-actin network. ARP2/3 was found in the branched actin suggesting the important role of ARP2/3 in strengthening and stabilizing the curved and branched actin structure. C-Src, cortactin, cofilin, and actin were found to accumulate around nuclei suggesting their role in nuclear processes such as the locomotion of the nuclei.

## Conclusions

The results show, with the increased precision provided by an STED microscopy, the distribution of subplasmalemmal actin. In addition, the results suggest a role for actin in clustering and perhaps translocation of osteoclast nuclei. These novel findings may pave the way for future studies on live cell imaging of various aspects of cellular movement, including cell migration, transcytosis, translocation of nuclei, and cell fusion.

## Electronic supplementary material

Below is the link to the electronic supplementary material.

The video shows the 3 –dimensional structure of adjacent osteoclasts and the bridging micrometer level tube between the cells. F-actin was visualized with phalloidin-Star635 staining (green) and the nucleus with PicoGreen staining (blue). The playback rate is 15frame/s. (MOV 743 KB)

## Abbreviations

STED: Stimulated emission depletion
TNT: Tunneling nanotubes
MLT: Micrometer-level tube
RANKL: Receptor activator of nuclear factor kappa-B ligand
M-CSF: Macrophage colony-stimulating factor
APD: Avalanche photo diode
PBS: Phosphate buffered saline
BSA: Bovine serum albumin
